# Confrontment and solution to gonadotropin resistance and low oocyte retrieval in in vitro fertilization for type I BPES: a case series with review of literature

**DOI:** 10.1186/s13048-021-00900-2

**Published:** 2021-10-28

**Authors:** Yiqi Yu, Mengxia Ji, Weihai Xu, Ling Zhang, Ming Qi, Jing Shu

**Affiliations:** 1 Department of Reproductive Endocrinology, Reproductive Medicine Center, Zhejiang Provincial People’s Hospital, Affiliated People’s Hospital, Hangzhou Medical College, Hangzhou, China; 2 Department of Cell Biology and Medical Genetics, Zhejiang University School of Medicine, Hangzhou, China

**Keywords:** “Infertility”, “Blepharophimosis, ptosis, and epicanthus inversus syndrome”, “In vitro fertilization”, “FOXL2”, “Gonadotropin resistance”

## Abstract

**Background:**

*FOXL2* mutations in human cause Blepharophimosis, ptosis, and epicanthus inversus syndrome (BPES). While type II BPES solely features eyelid abnormality, type I BPES involves not only eyelid but also ovary, leading to primary ovarian insufficiency (POI) and female infertility. Current mainstream reproductive option for type I BPES is embryo or oocyte donation. Attempts on assisted reproductive technology (ART) aiming biological parenthood in this population were sparse and mostly unsuccessful.

**Case presentation:**

Two Chinese type I BPES patients with low anti-müllerian hormone (AMH) and elevated follicle stimulating hormone (FSH) presented with primary infertility in their early 30s. Genetic studies confirmed two heterozygous duplication mutations that were never reported previously in East Asian populations. They received in vitro fertilization (IVF) treatment and both exhibited resistance to gonadotropin and difficulty in retrieving oocytes in repeated cycles. Doubled to quadrupled total gonadotropin doses were required to awaken follicular response. Patient 1 delivered a baby girl with the same eyelid phenotype and patient 2 had ongoing live intrauterine pregnancy at the time of manuscript submission.

**Conclusions:**

This is the second reported live birth of biological offspring in type I BPES patients, and first success using IVF techniques. It confirmed that ART is difficult but feasible in type I BPES. It further alerts clinicians and genetic counsellors to type female BPES patients with caution in view of the precious and potentially narrowed reproductive window.

**Supplementary Information:**

The online version contains supplementary material available at 10.1186/s13048-021-00900-2.

## Background

Blepharophimosis, ptosis, and epicanthus inversus syndrome (BPES) is a rare genetic disease involving predominantly the eyelid and ovary. The prevalence of this disease is estimated to be 1 in 50,000 births [1]. Interestingly, this pleiotropic effect results from a single gene mutation of *FOXL2* rather than from contiguous gene syndrome. 67% to over 80% patients were detected mutation-positive in *FOXL2* gene after clinical diagnosis based upon four typical eyelid features: blepharophimosis, ptosis, epicanthus inversus, and telecanthus [2, 3]. Patients with only eyelid features were classified into type II BPES, which was reported to almost always inherit in autosomal dominant pattern, with nearly complete penetrance. Description of the association of POI (Primary ovarian insufficiency)-related female infertility with eyelid features dates back to as early as 1983, almost two decades before the precise gene localization [4–6]. Patients with such phenotypic combination were classified into type I BPES.

Efforts are being made to specify the phenotype-genotype correlation in type I BPES, in order to predict ovarian risk and guide long-term follow-up strategy for affected females at an early age. Unfortunately, unlike the eyelid feature, variable interfamilial and intrafamilial expressivity of ovarian function and fertility was observed [7]. The onset of POI in an individual is neither confidently predictable nor therapeutically reversible at the moment. Current strategy for confirmed type I BPES primarily compromises of two stems: 1) gynecological management of POI by hormone replacement therapy (HRT); 2) Assistance in completing family. The mainstream options for the latter provided at genetic counseling renders biological parenthood, which includes donation of eggs or embryos, adoption or fostering. Precautionary ovarian tissue cryopreservation or oocyte cryopreservation for girls or unmarried women is neither widely used nor reported to be successful for type I BPES yet.

Here we describe two Chinese type I BPES patients with heterozygous *FOXL2* duplication mutations that were published for the first time in East Asian populations. The duplication in patient 2 has not been reported to have caused type I BPES previously. More importantly, it was the first reported attempt of IVF on type I BPES patients, which revealed characteristic profile including discordance between antral follicle count (AFC) and anti-müllerian hormone (AMH), gonadotropin resistance, low follicular output rate (FORT) as well as low oocyte retrieval rate (ORR). By sharing our challenge and success, we wish to provide heads-up, hope and help to BPES patients.

## Case presentation

### Clinical history

Both patients had typical eyelid features and had received plastic surgery in childhood. They presented to our clinic at the age of 32 for infertility. Patient 1 had menarche at the age of 12, maintaining overall regular menstrual cycle thereafter, except for one episode of menorrhagia which revealed complex hyperplasia and was treated with Mirena. She had a spontaneous pregnancy when she was 30 after actively trying to conceive for one and half years, but unfortunately miscarried at 7 weeks of gestational age. Patient 2 had menarche at the age of 13, followed by oligomenorrhea ever since. Ovulation induction with gonadotropins failed at the dose up to hMG 150 IU/day.

### Infertility work-up

On clinical examination, both patients exhibited well-developed secondary sexual characteristics. Body mass index (BMI), Tanner staging of pubic hair and breasts were summarized in Table 1. Both patients’ AMH and basal hormone profile implied decreased ovarian reserve, But ultrasound scan discordantly showed plenty bilateral antral follicles (Table 1). Their uteri were of normal size. For both patients, hysterosalpingogram and husband’s semen analysis were unremarkable [7].

**Table 1:** Details in physical examination and laboratory findings related to ovarian function

	Ht (cm)	Wt (kg)	BMI	Tanner staging	AMH (ng/mL)	bFSH (mIU/mL)	bLH (mIU/mL)	bE_2_ (pg/mL)	bP (ng/mL)	Testosterone (ng/mL)	Prolactin (ng/mL)	AFC ^a^ (RO/LO)
Patient 1	154	55	23.2	5	0.45-0.56	10.94-13.71	6.28-6.44	22.89-69.00	0.08-0.26	0.46-0.53	6.43-11.42	6-10+/ 6-8
Patient 2	155	65	27	5	1.0-1.59	5.25-14.37	2.33-8.29	<10-82.60	0.16-0.23	0.23	11.35	4-9/3-5

*Ht* Height, *Wt* Weight, *BMI* Body mass index, *AMH* Anti-müllerian hormone, *bFSH* Basal follicle stimulating hormone, *bLH* Basal luteinizing hormone, *bE*_*2*_ Basal estradiol, *bP* Basal progesterone, *AFC* Antral follicle count, *RO* Right ovary, *LO* Left ovary

Basal hormone profiles were summarized from different menstrual cycles across patients’ visits, illustrated using lowest and highest readings

^a^Measurements of AFC were done by experienced fertility specialists

### IVF treatment

Antral follicle count (AFC): number of follicles with diameter between 4milimetre (mm) to 9 mm seen at day 2–3 of menses.

Preovulatory follicle count (PFC): number of follicles with diameter between 14 mm to 22 mm.

Follicular output rate (FORT) = PFC/AFC × 100% [8].

Oocyte retrieval rate (ORR) = number of oocytes retrieved/PFC × 100% [9].

Both patients received two cycles of controlled ovarian hyperstimulation (COH) and obtained only one embryo. Patient 1 received first cycle in another centre and second cycle in our centre. Details and outcomes of COH in her first cycle were obtained from her outpatient medical record with authorization. Patient 2 received both cycles in our centre.

**Fig. 1:**
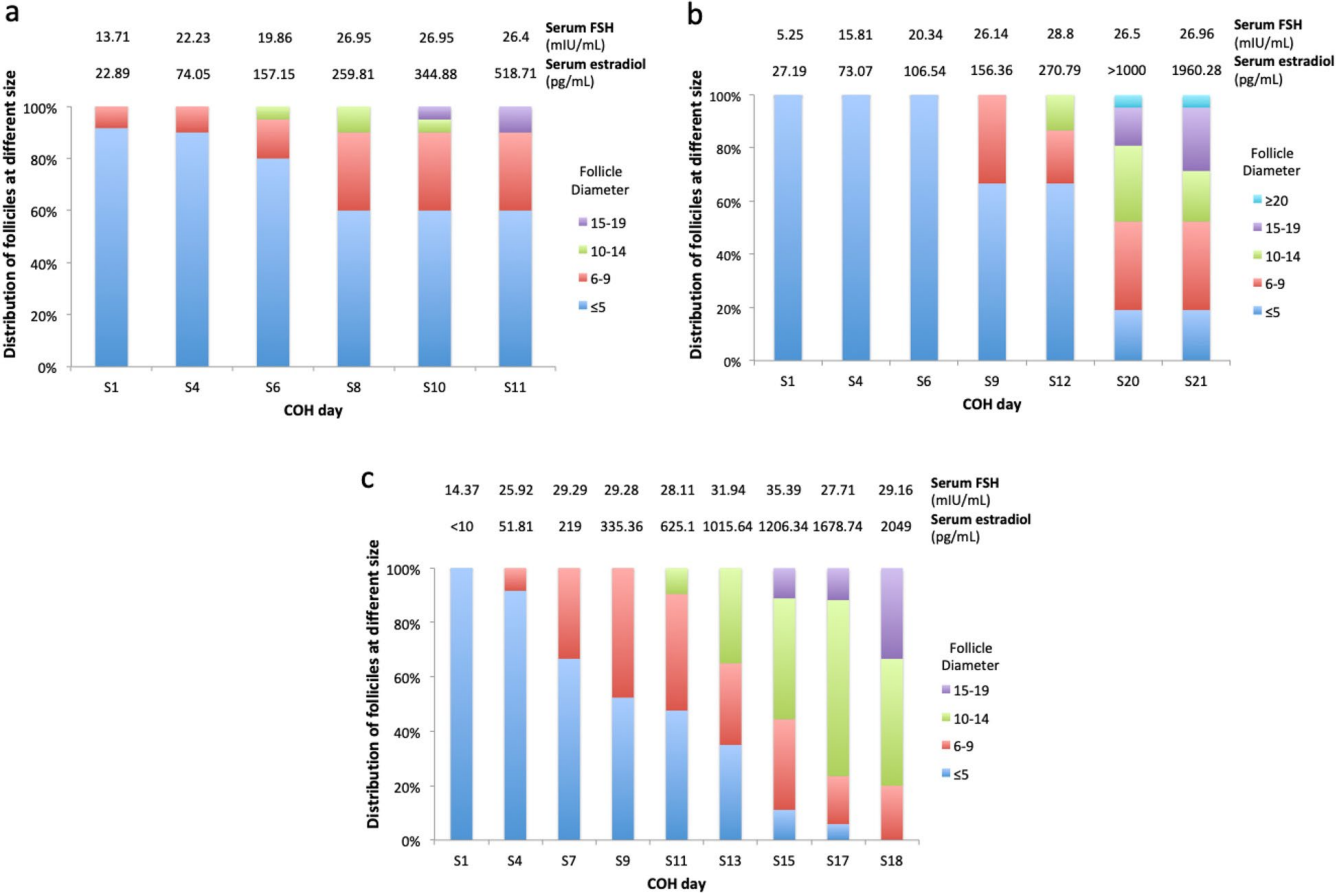
Follicular growth under controlled ovarian hyperstimulation (COH) showing the relationship between serum FSH level and follicular response as well as the corresponding serum estradiol level. Follicles are divided into 5 groups: ≤5 mm, 6-9 mm, 10-14 mm, 15-19 mm, ≥20 mm. Percentage of each group is shown. S1 = Day 1 of hyperstimulation. **a** Patient 1's second COH cycle; **b** Patient 2's first COH cycle, note that no exact estradiol reading was recorded on S20 because the corresponding serum sample was not diluted; **c** Patient 2's second COH cycle

In first COH cycle for patient 1, unexpected gonadotropin resistance was observed under GnRH agonist protocol, rendering her a long hyperstimulation course with increasing dose of hMG up to a maximum of 450 IU per day. Not one oocyte was harvested from the six preovulatory follicles. GnRH antagonist protocol was used for her second cycle in our centre. COH was started at a dose of rFSH 150 IU plus hMG 150 IU per day, and was increased to rFSH 150 IU plus hMG 300 IU per day at the sixth day of COH due to poor response (Fig. 1a). In view of previous oocyte retrieval failure, the oocyte retrieval time was postponed to 40 h post ovulation-triggering instead of the routine 36 h. two preovulatory follicles were punctured, but only one oocyte was harvested on first flushing using double-lumen needle. The FORT and ORR were 0.16 and 0.5 respectively despite doubled total gonadotropin dose, much lower than those of patients at the same age undergoing IVF in our centre (Table 2). This mature oocyte was normally fertilized into 2PN (2pronuclear) zygote following conventional IVF technique, and a blastocyst graded 5 AC was frozen.

**Table 2:** Details in controlled ovarian hyper-stimulation (COH) and oocyte retrieval in IVF

	AFC	PFC	OR	FORT	ORR	Total Gn dose (IU)	Gn/day (IU)	COH days	Gn/Weight (IU/kg)	Ovulation triggering medication /ovulation triggering-to-harvest interval
Patient 1 Cycle 1 (at 32yo) ^a^	N/A	6	0	N/A	0	N/A	N/A	N/A	N/A	N/A / 36hours
Patient 1 Cycle 2 (at 32yo)	12	2	1	0.16	0.50	4050	368	11	73	GnRH agonist 0.1mg+rhCG 250mcg / 40hours
Patient 2 Cycle 1 (at32yo)	13	7	1	0.54	0.14	9600^c^	457	21	147	GnRH agonist 0.1mg+rhCG 250mcg / 36hours
Patient 2 Cycle 2 (at 32yo)	7	7	3	1.00	0.42	8850^c^	491	18	136	GnRH agonist 0.1mg+rhCG 250mcg / 36hours
Controls ^b^	8.2	6.5	6.8	0.91	1.03	2362	254	9.4	43	Varied

*AFC* Antral follicle count, *PFC* Preovulatory follicle count, number of follicles ≥14mm at triggering day, *OR* Number of oocytes retrieved, *FORT* Follicular output rate = PFC/AFC ×100%, *ORR* Oocyte retrieval rate = number of OR/PFC ×100%, *Gn* Gonadotropin

^a^ Patient 1 cycle 1 was done at another centre, and therefore some clinical data was not available

^b^Averaged data from our IVF centre, calculated from all cycles done in the same year for same-aged patients

^c^Clomiphene citrate was used simultaneously

In first COH cycle for patient 2, combined use of hMG and clomiphene citrate was administrated simultaneously at a starting dose of 225 IU/day and 100 mg/day from day 3 of menses. Both ovaries were barely responsive to serum FSH at 20 mIU/mL achieved by hMG stepped-up to 450 IU/day. HMG was eventually increased to 525 IU/day, and serum FSH reached 28.8 mIU/mL (Fig. 1b). After a total of 21 days of hyper-stimulation with a cumulative dose of gonadotropin at 9600 IU and clomiphene citrate at 2100 mg, seven preovulatory follicles developed but only one oocyte was harvested. This mature oocyte was normally fertilized following conventional IVF technique, but embryo development unfortunately arrested on day 2. A similar protocol was used in her second cycle, taking up 18 days and a cumulative dose of hMG at 8850 IU plus clomiphene citrate at 2300 mg. simultaneous use of hMG at 450 IU/day plus clomiphene citrate at 100 mg/day was started from day 3 of menses. HMG was increased to 525 IU/day and clomiphene citrate increased to 150 mg/day at COH day 9 due to poor response. Resultant serum FSH ranged from 25 to 35 mIU/mL (Fig. 1c). Three oocytes were harvested from seven preovulatory follicles, and were all mature. After conventional IVF, one 2PN zygote developed into a Grade-2 7-cell Cleavage-stage embryo with 5% fragmentation. The rest two oocytes ended up unfertilized (0PN) and abnormally fertilized (3PN) respectively. For both cycles the ovulation triggering-to-harvesting interval was 36 h, and oocytes were retrieved using double lumen needles on one to three times of flushing. The FORT and ORR were low, but improvement was seen in second cycle (Table 2).

### Genetic analysis

For each participant, genomic DNA was extracted from whole blood samples. High throughput target region sequencing (TRS) was carried out to cover all exons and ± 10 bp of neighboring introns, with depth at 122.15× and average coverage at 99.36%. All candidate variants were annotated and filtered against public databases including 1000 Genome, gnomAD, ClinVar, dbNSFP, LOVD and ZJU-DB. PCR and Sanger sequencing was performed to confirm variants within patients and other members in the family. In-silico analysis with the online tool PROVEAN was implemented. Mutation nomenclature was made according to Human Genome Variation Society (HGVS). Integrated interpretation was made according to American College of Medical Genetics and Genomics (ACMG) guideline.

**Fig. 2:**
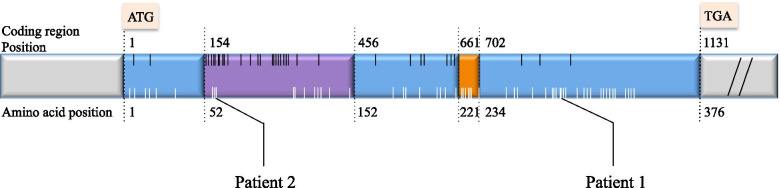
Positions of mutations detected in the two patients. Purple region: forkhead domain (FHD); Orange region: polyalanine tract (PAT); Blue region: the rest of coding region. Pathogenic or likely-pathogenic mutations submitted in ClinVar were summarized, presenting as short vertical lines: black line = point mutation; white line = mutation resulting in deletion or duplication

**Fig. 3:**
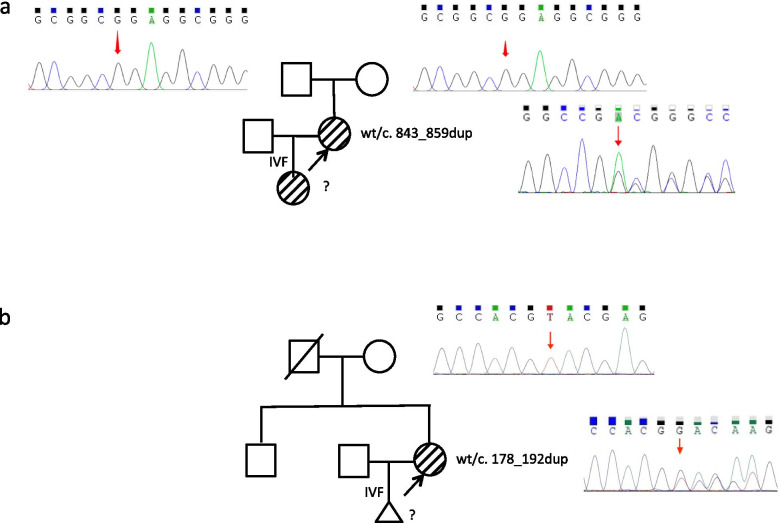
Pedigrees and Sanger sequencing of patients and their relatives. **a** Sanger sequencing for patient 1 and her parents is demonstrated. **b** Sanger sequencing for patient 2 and her mother is demonstrated

For patient 1, genetic testing revealed a de novo *FOXL2* duplication mutation (c.843_859dupGGCCGCACCCCCGCCTC) resulting in frameshift and premature termination (Fig. 2, Additional file 1). For patient 2, genetic testing uncovered a *FOXL2* duplication mutation (c.178_192dupGGCGATGAGCGCCAC) resulting in insertion of five hydrophobic amino acids, Val-Ala-Leu-Ile-Ala, between the 64th and 65th amino acids (Fig. 2, Additional file 1). It was likely a de novo mutation in view that neither eyelid abnormality nor infertility was reported in her family (Fig. 3b). Both couples opted not for preimplantation genetic testing (PGT) after thorough genetic counseling.

### Pregnancy outcomes

The frozen-thawed blastocyst of patient 1 was transferred after hormonal replacement preparation of endometrium. Pregnancy was confirmed on Day 9 post embryo transfer, and carried on uneventfully until 33 weeks of gestational age, when patient 1 was hospitalized for antepartum bleeding and shortened cervix. She delivered a baby girl at 36 weeks by caesarean section after prolonged preterm premature rupture of membrane (PPROM). The baby weighed 2600 g at birth, and manifested the same eyelid features as her mother (picture see “Additional file 2”).

The frozen-thawed Cleavage-stage embryo of patient 2 was transferred after hormonal replacement preparation of endometrium. Pregnancy was confirmed on Day 9 post embryo transfer, and latest follow-up at the time of manuscript submission confirmed ongoing single live pregnancy at 29 weeks of gestation.

### Literature search

We searched the PubMed database using search terms for Medical Subject Headings and/or text words relating to FOXL2 or BPES. The retrieved articles were further hand-searched using our inclusion criteria (papers reporting pregnancy in type I BPES). Only 1 case of type I BPES was reported to have achieved live birth by AID with monofollicular ovulation induced by high dose of rFSH (Table 3). No natural pregnancy was reported.

**Table 3:** Reported pregnant cases in type I BPES patients

Number	Ethnicity	Age	FOXL2 mutation	Amino acid changes	Clinical findings	Hormone status	Fertility therapy	Pregnancy outcome	Follow up	Reference
1	N/A	30	Genetic finding not given	N/A	Eyelid abnormality, oligomenorrhea, night sweats, Gn resistance	FSH 3.7-27.8mIU/mL, AMH 0.1-0.3ng/ml	AID with monofollicular ovulation was achieved by 14-day course of rFSH at 300IU/day.	Complicated by PTL and PE. Live DCDA twin births at 35 weeks	Patient on OCP after 1month breastfeeding. One twin had eye changes	Roth and Alvero, 2014
2	Chinese	32	c.843_859dupGGCCGCACCCCCGCCTC	p.Pro287ArgfsTer75	Eyelid abnormality, infertility, Gn resistance	FSH 10.94-13.7mIU/mL, AMH 0.45-0.56ng/ml	Conventional IVF	Complicated by APH, PPROM and PTL. Live birth at 33 weeks	Breastfeeding for more than 1 year. Baby girl had eyelid changes	Patient 1 in this article
3	Chinese	32	c.178_192dupGGCGATGAGCGCCAC	p.Val60_Ala64dup	Eyelid abnormality, oligomenorrhea, infertility, Gn resistance	FSH 5.25-14.37mIU/mL, AMH 1.0-1.59ng/ml	Conventional IVF	Ongoing pregnancy over 29weeks	N/A	Patient 2 in this article

*Gn* Gonadotropins, *AID* Artificial insemination with donor sperm, *PTL* Preterm labor, *PE* Preeclampsia, *DCDA* Dichorionic diamniotic, *OCP* Oral contraceptive pills, *APH* Antepartum hemorrhage, *PPROM* Preterm premature rupture of membrane

## Discussion

*FOXL2* gene belongs to the winged-helix/forkhead transcription factor family, members of which are highly conservative. Pathogenic variants lead to either disordered subcellular distribution or dysregulation of downstream genes [10]. Apart from the eyelid, *FOXL2* is most abundantly expressed in the ovary [11, 12]. It is an earliest granulosa cell marker, and plays a crucial role in sex determination, tumorigenesis, ovarian somatic cell proliferation and differentiation [13].

Both of the two patients presented to our reproductive medicine clinic for infertility with underlying ovarian function defect. Since their eyelid abnormality occurred early at birth and were surgically corrected before good understanding of the relationship between *FOXL2* mutation and BPES [4], in addition to the absence of family history, genetic diagnosis was delayed until adulthood.

During their fertility treatment, they were clinically resistant to gonadotropins, but not completely unresponsive. Furthermore, both exhibited paradoxically normal AFC despite low AMH, and thus did not fall into any of the four groups under Poseidon criteria [14]. Beyond that, both experienced poor ORR in repeated IVF cycles despite multiple flushing [15], conform to poor ovarian responders according to Bologna criteria [16]. In spite of all these difficulties, they were lucky to have succeeded with their one and only embryo.

This is inspiring news considering the very limited successes reported in biological parenthood achieved by assisted reproductive technology (ART) for BPES patients (Table 3). In a large cohort of 164 probands, a 31-year-old BPES patient (c.672_701dup) without hypergonadotropic hypogonadism was mentioned to have achieved pregnancy after IVF treatment, but indication for IVF was not specified [17]. A case report in 2014 described a 30-year-old nulligravid woman with clinically diagnosed type I BPES, who delivered twins after artificial insemination with donor sperm (AID) [18]. Similar ovarian resistance as our patients was observed during her gonadotropin stimulation, and follicles were unresponsive to rFSH at 125 IU/day. Monofollicular ovulation was achieved by 14-day course of rFSH at 300 IU/day. Although genetic evidence was not provided in that case report, typical eyelid changes in one twin seemed to support the presence of genetic inheritance, probably *FOXL2* mutation [3].

In patient 1 (c.843_859dup), insertion of 17 nucleotides (Fig. 2) resulted in frameshift in open reading frame (ORF) and a protein truncated after polyalanine tract (PAT), which belonged to group D according to classification suggested by De Baere E et al. [19]. It was a mutational hotspot responsible for approximately 13–18% of all intragenic pathogenic variants [17, 20], but was reported for the first time here in East Asian populations. Both type II BPES phenotypes with naturally completed family and type I BPES phenotypes had been observed in pedigrees with this mutational variant, revealing phenotypic variability and even variable intrafamilial expressivity of POI [17].

The rarer c.178_192 duplication found in patient 2 leads to an elongated protein. Noteworthily, the insertion of five hydrophobic amino acids occurs in the regulatory forkhead domain (FHD) (Fig. 2). Studies have shown that missense mutations in the FHD impaired not only subcellular localization but also transactivation activity of *FOXL2*, with either loss-of-function or dominant- negative effect [21]. FHD-deleted *FOXL2* mutants lost the direct binding ability to SF-1 in vitro, which mediated its repression on steroidogenic genes *CYP17* [22]. Elongation from inside the FHD was not included in the classification suggested by De Baere E *et a l.* [19], but considering that it occurred immediately after the start of this functional domain, deranged protein function in regulatory activity was suspected. Clinically patient 2 manifested more severe phenotype with oligomenorrhea. Ovarian resistance was also stronger, even after adjustion for body weight. First described pedigree with this duplication was a mother and son with BPES eyelid features. The mother reported oligomenorrhea and menorrhagia. Ovarian function and age of reproduction was not mentioned. Since there was absence of POI and infertility history, the mother was grouped into type II BPES. Second case was a British boy aged 8 in 2019 [2], but family history was not stated. To our knowledge, this mutation was never reported in East Asian populations, and never reported to have caused classic type I BPES phenotype.

Further studies are needed to elucidate the pathophysiology of the distinctive ovarian characteristics in these *FOXL2* mutants:

1. Actual follicular reserve behind the discordance between AFC and AMH: Considering the relative abundance of gonadotropin-responsive (even though resistant) antral follicles in both of our patients at their age, the mechanism of low AMH in type I BPES patients may differ from patients with non-genetic causes of ovarian insufficiency, such as advanced age, or acquired pathology (e.g endometriosis, surgery

, or chemotherapy) [23]. AMH is produced in primordial follicles, preantral follicles and small antral follicles [24]. It works as an inhibitor of upstream primordial follicle recruitment [25]. FOXL2 works within a broader span of time along the follicular development, from multipotential stromal cells to cumulus cells in preovulatory follicles [13]. It was speculated that the mutual regulation of the two genes formed a positive feedback loop to maintain ovarian reserve [26]. Small follicles expressing mutant *FOXL2* may have lowered expression of *AMH*, leading to the paradoxy.

Without the guarding by properly-expressed *AMH* and *FOXL2* at different stages, is there imminently exhausted primordial follicle pool behind the ultrasonographic normal AFC? We do not have ovarian tissue histology to uncover the real situation in our two patients, but previous studies suggested risk of follicular depletion at an alarming rate, which eventually led to the well-known final stage of POI in type I BPES. Two siblings with a strong family history of autosomal dominant inheritance of BPES were reported in 1988, before establishment of genetic causation. The elder sister aged 28 and the younger sister aged 20 presented with secondary amenorrhea and hypergonadotropic hypogonadism. Ovarian pathological sections obtained from laparoscopy respectively showed true premature menopause with total absence of primordial follicles in the elder sister, and normal number of primordial follicles in the younger sister [27]. In *Foxl2*^*lacZ*^ homozygous murine ovaries, the presence of defected squamous-to-cuboidal transition and premature expression of Gdf9 indicated accelerated depletion of primordial follicles and oocytes atresia, providing plausible explanation for the elder sister’s histological finding. In another paper, two patients with *FOXL2* deletions after PAT and resultant putative elongated proteins were reported. The 19-year-old patient suffered from primary amenorrhea with underlying low AMH and abnormal ovarian histology (polyovular follicles, defective basal lamina, deposition of cholesterol crystals, advanced expression of proliferative marker Ki67, and intracytoplasmic *FOXL2* aggregation). The 25-year-old patient presented with infertility, but had normal AMH and normal ovarian reserve clinically, ultrasonographically, and histologically. But there was also evidence of uncommon advanced expression of Ki67 in transitional follicles implying accelerated recruitment [28].

2) Gonadotropin resistance: Our patients were resistant to gonadotropins, yet responsive at high doses with extended courses. Target serum FSH seemed to be higher than 25 to 30 mIU/mL. Doubled to quadrupled total gonadotropin doses in comparison to general population was required in various COH protocols in order to awaken follicular response, and clomiphene citrate was added to patient 2 for enhancement.

Clomiphene citrate is a selective estrogen receptor modulator. It was utilized in ovulation induction or stimulation by preventing estrogen negative feedback and thereby stimulating endogenous gonadotropin release. It was shown that supplement of clomiphene improved ovarian stimulation outcomes in IVF treatment and decreased total gonadotropin dosage, which applied to women with advanced age and/or poor response [29, 30]. We adopted the combined use of clomiphene citrate and gonadotropin into the COH treatment for our two type I BPES patients. Both of the patients were on the road of accelerated ovarian function decline in addition to gonadotropin resistance. However, unlike patients with POI or imminent POI due to advance age or iatrogenic cause, supraphysiological level of serum FSH level seemed to correlate positively to ovarian response and IVF outcome.

As a result of the augmentation of hyperstimulation, FORTs could be improved but still low compared to general IVF patients. Apart from the AID case mentioned above, who succeeded by using high dose rFSH, few case reports, before and after *FOXL2* gene localization, also described the phenomenon of ovarian resistance to gonadotropins in BPES patients [27, 31–34], but failed either in ovulation induction or in conception. Is the resistance universal to type I BPES patients? Is it the major cause of infertility? Answers are not clear yet. We speculate that the genes involved in FSH responsiveness and/or antral follicle growth, such as FSH receptor (FSHR), might be dysregulated in mutants through direct or indirect mechanisms.

3) Abnormal ovulation and low ORR: Less studies focused on ovulation in BPES patients. As described, patient 2 was oligomenorrheic secondary to anovulation. Patient 1 maintained overall natural normal cycle with sonographically confirmed ovulation. It was at least once followed by successful natural fertilization and implantation but unluckily ended up with first-trimester miscarriage. However, she also reported to have experienced cycles judged as luteinized unruptured follicle syndrome (LUFS). Furthermore, the history of endometrial hyperplasia implied possible preceding anovulation. There might be some mechanism behind the disordered ovulation of graafian (preovulatory) follicles as well as the difficulty in harvesting oocytes from them in IVF. Hypotheses include hampered oocyte maturation, abnormal expression of Luteinizing hormone receptor (LHR) [35], defected gap junction, and deranged activity of local factors such as prostaglandin and proteases [36]. Individualized ovulation triggering-to-harvesting interval and two-chamber needle flushing may have a role in improving the ORR. In view of the likely innate gonadotropin resistance, an increased dose of LH/hCG dosage for triggering, which was not tried in our cases, may also contribute potential benefit.

Currently mainstream recommendation at genetic counseling for type I BPES patients includes donation of eggs or embryos, adoption or fostering, sacrificing biological parenthood. This is probably based on the following considerations: 1) missed reproductive window at presentation; 2) limited translation of in vitro maturation (IVM) into clinical use; 3) ethical concerns about precautionary ovarian tissue cryopreservation or oocyte cryopreservation for young girls with unclear ovarian phenotype; 4) risk of inheritance of pathogenic mutations.

Noteworthily on the other hand, patients manifesting either type of BPES usually have normal menarche and secondary sex characteristics. Association with major defects including intellectual disability were rare with inconclusive causation. Even type I BPES phenotypes with primary or secondary amenorrhea possess potential capacity of natural conception and deserve a childbearing opportunity. Early intervention may rescue their threatened fertility.

Clinicians and scientists were making efforts to specify the phenotype-genotype correlation in ovarian function [19]. Studies suggested high POI risk from mutations with proteins truncated upstream of PAT and from mutations involving inward-pointing of relevant amino acids towards the hydrophobic core. Meanwhile PAT expansion predominantly leads to type II BPES [10]. However, considering the inter-individual variability with even varied intrafamilial penetrance of ovarian phenotypes from identical genotypes, POI risk should be emphasized to all female patients as early as possible while an efficient prediction model is still on the way. As the putative acceleration of follicular depletion takes place without clear red flag for onset and exhaustion, it is unrational to declare innocence and passively wait till the age of 40 to diagnose POI, especially considering the much higher difficulty they might encounter like our patients, compared to patients with other causes of low ovarian reserve.

In summary, we described detailed IVF treatment to two Chinese type I BPES patients, whose *FOXL2* mutations were new to East Asian population database. Their ovarian profiles seem to differentiate type I BEPS from either pure resistant ovarian syndrome (ROS) or pure POI, and offers materials for further basic research on *FOXL2* gene and type I BPES. Of course, due to the rarity of the disease, we look forward to more cases to reproduce the success and the observed phenomena. Nevertheless, our report confirmed that IVF in type I BPES is difficult, but still feasible with individualized treatment. It also serves as a stepping-stone to future attempts on IVM and PGT in this population.

## Supplementary Information

**Additional file 1.** Summary of genetic analysis findings (table).

**Additional file 2.** Patient 1’s daughter-facial manifestations.

## Data Availability

The datasets supporting the conclusions of this article are included within the article (and its supplementary files).

## Abbreviations

AFC: Antral follicle count
AID: Artificial insemination with donor sperm
AMH: Anti-müllerian hormone
ART: Assisted reproductive technology
BPES: Blepharophimosis, ptosis, and epicanthus inversus syndrome
COH: Controlled ovarian hyperstimulation
FORT: Follicular output rate
FSH: Follicle stimulating hormone
GnRH: Gonadotropin-releasing hormone
hMG: Human menopausal gonadotropin
IVF: In vitro fertilization
LH: Luteinizing Hormone
ORR: Oocyte retrieval rate
PGT: Preimplantation genetic testing
POI: Primary ovarian insufficiency
PN: Pronuclear
ROS: Resistant ovarian syndrome
