# Supplementary material for: Confrontment and solution to gonadotropin resistance and low oocyte retrieval in in vitro fertilization for type I BPES: a case series with review of literature
Source: J Ovarian Res. 2021 Oct 28;14:143. doi: 10.1186/s13048-021-00900-2 (PMC8555206; doi:10.1186/s13048-021-00900-2)
Supplement: Supplementary file 1 — Additional file 1. Summary of genetic analysis findings (table). [file 13048_2021_900_MOESM1_ESM.docx]

**Supplementary Table 1** Summary of genetic analysis findings

| **Proband** | **Mutation** | **Amino acid change** | **Parents** | **PROVEAN Score** | **gnomAD exome EAS** | **Phenotype** | **Previously reported phenotypes** | **ACMG classification of Pathogenicity** |
| --- | --- | --- | --- | --- | --- | --- | --- | --- |
| Patient 1 | c.843_859dupGGCCGCACCCCCGCCTC | p.Pro287ArgfsTer75 | Mother WT  Father WT | Frameshift | N/A | Type I BPES | Type I BPES, type II BPES | Pathogenic |
| Patient 2 | c.178_192dupGGCGATGAGCGCCAC | p.Val60_Ala64dup | Mother WT  Father unknown^a^ | -13.59(Deleterious) | N/A | Type I BPES | Type II BPES | Likely pathogenic |

gnomAD exome EAS: Genome aggregation database for East Asian population. ACMG: American College of Medical Genetics Classification criteria. WT: Wild type.

^a^ Patient 2’s father was deceased but was reported to have no eyelid abnormality
