# Supplementary figures and images for: Confrontment and solution to gonadotropin resistance and low oocyte retrieval in in vitro fertilization for type I BPES: a case series with review of literature
Source: J Ovarian Res. 2021 Oct 28;14:143. doi: 10.1186/s13048-021-00900-2 (PMC8555206; doi:10.1186/s13048-021-00900-2)

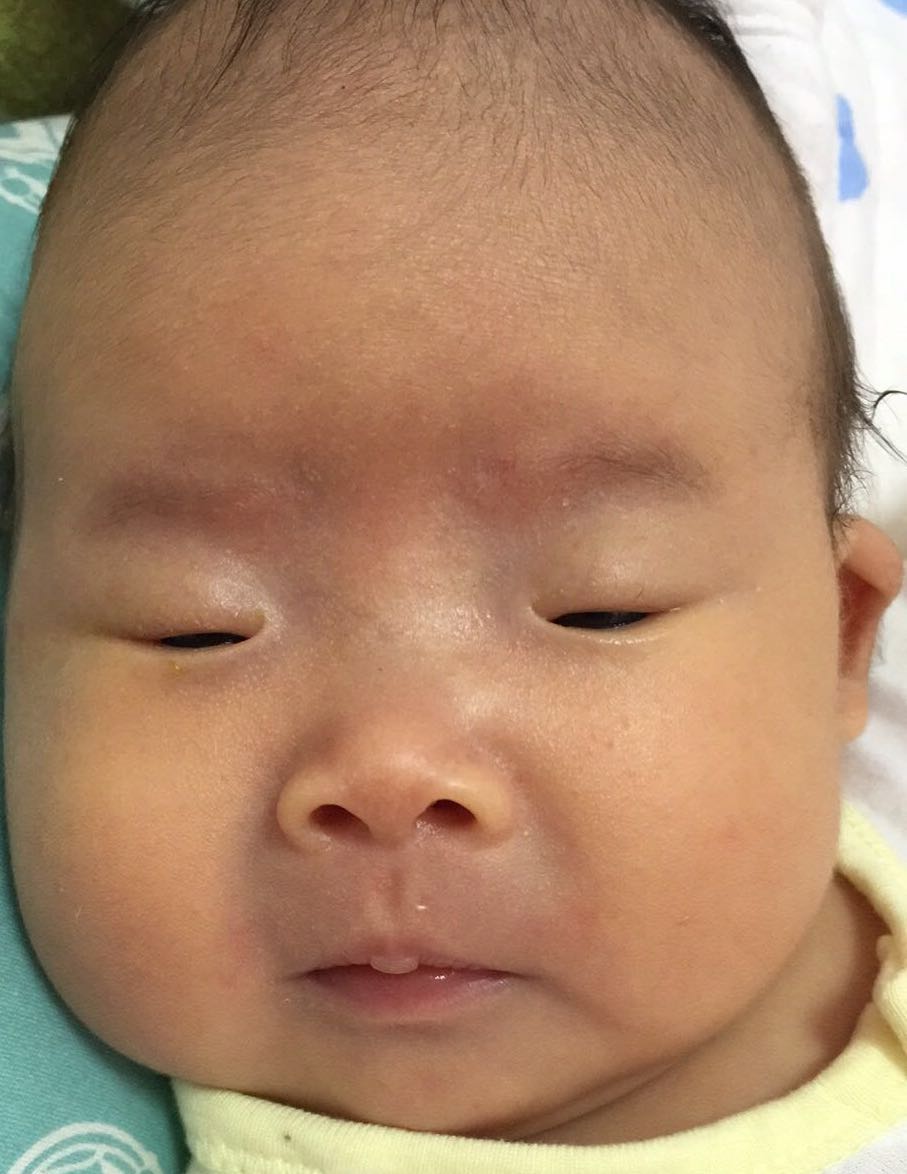

Supplement: Supplementary file 2 — Additional file 2. Patient 1’s daughter-facial manifestations. [file 13048_2021_900_MOESM2_ESM.jpg]
